# An Ontology-Based Cybersecurity Framework for the Internet of Things

**DOI:** 10.3390/s18093053

**Published:** 2018-09-12

**Authors:** Bruno Augusti Mozzaquatro, Carlos Agostinho, Diogo Goncalves, João Martins, Ricardo Jardim-Goncalves

**Affiliations:** 1 Faculdade de Ciências e Tecnologia, Universidade NOVA de Lisboa, 2829-516 Caparica, Portugal; jf.martins@fct.unl.pt (J.M.); rg@uninova.pt (R.J.-G.); 2 Centre of Technology and Systems, UNINOVA, 2829-516 Caparica, Portugal; ca@uninova.pt; 3 University of Surrey, 388 Stag Hill, Guildford GU2 7XH, UK; diogojg15@gmail.com

**Keywords:** cybersecurity framework, Internet of Things, security ontology, security service provisioning, Industry 4.0

## Abstract

The use of sensors and actuators as a form of controlling cyber-physical systems in resource networks has been integrated and referred to as the Internet of Things (IoT). However, the connectivity of many stand-alone IoT systems through the Internet introduces numerous cybersecurity challenges as sensitive information is prone to be exposed to malicious users. This paper focuses on the improvement of IoT cybersecurity from an ontological analysis, proposing appropriate security services adapted to the threats. The authors propose an ontology-based cybersecurity framework using knowledge reasoning for IoT, composed of two approaches: (1) design time, which provides a dynamic method to build security services through the application of a model-driven methodology considering the existing enterprise processes; and (2) run time, which involves monitoring the IoT environment, classifying threats and vulnerabilities, and actuating in the environment ensuring the correct adaptation of the existing services. Two validation approaches demonstrate the feasibility of our concept. This entails an ontology assessment and a case study with an industrial implementation.

## 1. Introduction

The term Internet of Things (IoT) became popular in the late 1990s after having several technologies associated with sensor development and machine control, connected to the World Wide Web [1]. However, recent developments in wireless sensor networks and Industry 4.0 motivated the expansion of IoT applications to different domains such as the industrial Internet, smart cities, the smart grid and healthcare [2,3,4,5,6]. Manyika [7] argues that IoT technologies have the ability to reach a total economic value of $11.1 trillion by 2025, a value that is equivalent to approximately 11% of the world economy. As an increase in the adoption of Industry 4.0 arises, productivity will boost amongst the manufacturing sectors. Several efforts have been made, which seek an automated cyber-physical interconnection between virtual and physical worlds, correlating data from the industrial shop floors with run time feedback from the systems. It intends to optimize production, resulting in new hybrid business models and exploiting intelligent technologies whilst accelerating innovation cycles. Cyber-Physical Systems (CPS), which are closely tangled with the IoT implementation, refer to the use of physical components (e.g., sensors) to gather data, to process them and used them in the cyber world [8]. CPS has affected society with provisioning of the interacting networks of the physical and computational equipment of smart services.

The origin of IoT has been attributed to Kevin Ashton [9], co-founder of the Auto-ID Centre at MIT. It is defined as uniquely identifiable things connected to form a dynamic worldwide network through sensors and a platform with the potential to improve everyday activities of our lives. According to Chase [10], “The IoT creates an intelligent, invisible network fabric that can be sensed, controlled and programmed. IoT-enabled products employ embedded technology that allows them to communicate, directly or indirectly, with each other or the Internet”. This scope allows for the development of new services and applications to connect smart objects, integrating network technologies, devices, sensors, software and distinct infrastructures to build new businesses and services.

### 1.1. The Central Challenge: IoT Cybersecurity

One of the most important aspects regarding the complete adoption of the Internet of Things in the real world is cybersecurity [11]. The heterogeneous connectivity of numerous IoT systems conveys several challenges and possible threats. Indeed, the protection of the IoT increases the task for security experts since it involves security provisioning services to billions of smart objects.

**Figure 1:**
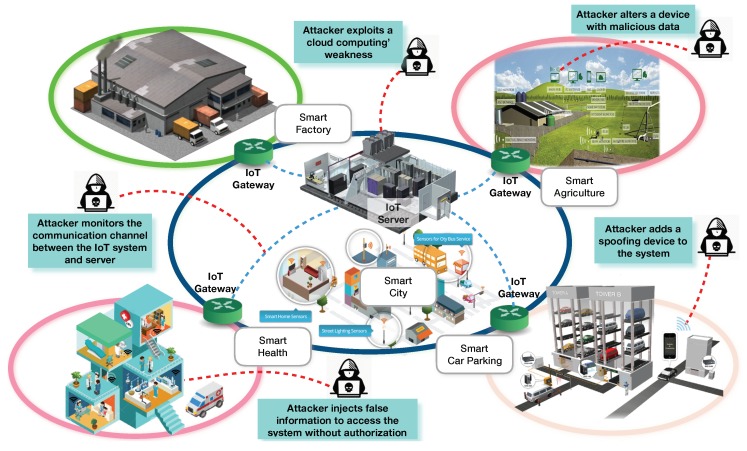
Potential security challenges for the IoT ecosystem.

The high number of incidents with IoT technologies is one of the main challenges that must be addressed when discussing the future of cybersecurity. As illustrated in Figure 1, there are several threats present within IoT systems such as spoofing, traffic sniffing, manipulation of sensitive information, code injections, unauthorised access, and so forth. Attacks can occur at different points in the IoT system, which stresses the importance of cybersecurity. According to Wolf and Serpanos [12], these systems must be designed and operated under a unified view of safety and security characteristics because they deal with the physical world and many times with critical activities.

IoT cybersecurity involves the inherent complexity of the IoT, which is further aggravated by the multiple heterogeneous exchanges of information, occurring between IoT devices and systems. Such devices tend to be increasingly exposed to the World Wide Web, which poses an ever-changing risk of new threats and undiscovered vulnerabilities [13].

Another challenge within the IoT ecosystem involves the lack of knowledge of the basic elements of cybersecurity: assets, threats, security mechanisms, vulnerabilities and security properties. Different IoT systems require distinct security mechanisms to avoid intrusions from the physical and cyber world. In other words, a compromised IoT device can be looked upon as an entry point for malicious users to gather sensitive user information, which results in the loss of two security properties: integrity and confidentiality. Therefore, the concept of IoT cybersecurity can become critical, affecting the adoption of IoT in multiple domains. Indeed, a 2016 survey conducted by Dell found that cybersecurity professionals were 49% more likely to spend additional time securing their data with sufficient information if they correctly understood the risks and threats faced by cybersecurity [14].

There are several traditional security mechanisms and services to mitigate specific threats. Despite this, the use of intelligent IoT systems allows for data to be gathered from sensors (e.g., network probes) and consequently processed disregarding those mechanisms. IoT devices and sensors are often deployed to perform functions without accounting for the presence of possible vulnerabilities, hence becoming susceptible to eavesdropping, jamming, tampering, jamming, etc.

These aforementioned potential security vulnerabilities have a substantial impact on IoT environments. To deal with these threats to IoT cybersecurity, our proposal follows an example that will be used along the paper, illustrating the solutions proposed: “An IoT environment is frequently susceptible to WiFi threats and easily targeted due to misconfigured access points, data interception, and denial of service. The use of a weak cryptographic algorithm without cryptographic integrity protection, as in the case of the Wired Equivalent Privacy (WEP) [15], compromises Wireless Local Area Network (WLAN) security”. Our vision is to present a novel approach to improve IoT cybersecurity using network and process monitoring for identifying and classifying vulnerabilities in a knowledge base and applying suitable security services adjusted to the particular threats.

### 1.2. Hypothesis and Main Results

As new wireless technologies are adopted, new exploits are appearing in the IoT ecosystem with a prevalent focus on increasing the exposition of data to potential threats. As a result, there must be greater knowledge of the elements in IoT systems to understand correctly how the prevalent cybersecurity issues at hand can potentially impact the function of the latter. As depicted in Figure 1, some threats try to manipulate data, access sensitive information and/or monitor communication channels. Therefore, the authors claim that “If knowledge about known cybersecurity issues (e.g., vulnerabilities, known threats), and the corresponding prevention measures could be integrated in a comprehensive ontology that is accessible to run time monitoring and actuation tools, then security systems could be improved to automatically detect threats to the IoT network and dynamically propose or implement suitable protection services.” To verify this hypothesis and improve IoT cybersecurity on the basis of the aforementioned problems, this paper presents an ontology-based cybersecurity framework, and the main contributions of this paper are listed below:

- The cybersecurity framework itself: an integrated technical framework using ontology and knowledge reasoning to address the security aspect of the Internet of Things within industrial environments. The framework focuses on the enterprise (company-side) monitoring, security analysis and the subsequent security service design and provisioning to improve business processes and technology assets;
- The IoTSec ontology, which is a core-component of the framework and a continued work of the authors (see [16]), gathering cybersecurity knowledge about alerts and possible threats and providing reasoning capabilities to discover implicit data from the contextual information of security issues;
- Design and orchestration method to implement and provide suitable security services in the IoT environments through the application of the Model-Driven Service Engineering Architecture (MDSEA) methodology (see [17]);
- Runtime security monitoring and actuation services integrated with the IDMEF standard (see [18]).

The rest of the paper is organized as follows: Section 2 presents related works. Emphasis is placed on cybersecurity frameworks and methods for service developments. With a focus on cybersecurity issues, our ontology-based cybersecurity framework is proposed in Section 3. Next, Section 4 presents the validation of our proposal considering two viewpoints. An ontology assessment to identify weaknesses and strengths is made, and a case study of an industrial scenario is presented. Final considerations from this paper and a roadmap on research directions for future works are outlined in Section 5.

## 2. Related Works

The literature analysis conducted suggests that there are several initiatives to provide cybersecurity for IoT systems, predominantly through the use of frameworks [19,20,21,22,23].

Ficco [19] proposed a hybrid and hierarchical event correlation approach for intrusion detection in cloud computing. The author provided a complex event analysis supported by an ontology to detect intrusion symptoms in a distributed approach. It gathers symptoms to report if a certain action is a successful attack or a non-relevant behaviour. A complex query analysing a sequence of requirement conditions is performed on the knowledge base to decide whether the particular behaviour represents a potential threat. The same author has also explored this proposal as a distributed intrusion detection in Cloud environments [20]. However, our proposed solution uses the ontology not for intrusion detection, but for vulnerability and threat analysis and as a support for service design and provisioning to prevent or recover from potential attacks.

Alam et al. [21] proposed a layered architecture of IoT to provide secure access provisioning to IoT-enabled things and interoperability of security attributes between distinct administrative domains. They used a semantically enhanced overlay to interlink layers, in which the ontology reasoning and semantic rules enabled the security aspects in a machine-to-machine platform. However, the authors only focused on security requirements of the access control issues, i.e., the semantic rules were designed to ensure access authorization. In contrast, our work can identify and provide security services using the ontology, with reasoning and querying capabilities.

The authors Tao et al. [22] proposed an ontology-based security service framework for IoT-based smart homes handling heterogeneity issues such as security and privacy preservation in a novel multi-layer cloud architectural model and enabling interactions on heterogeneous devices/services. The authors adopted ontologies to model and describe the different aspects of the IoT resources and a security ontology to achieve the security and privacy preservation in the process of interactions. However, the authors designed a small ontology considering only security properties (integrity and confidentiality) and the key carrier (security token) in the process of interactions. They did not explore the reasoning capabilities to infer implicit knowledge on the security ontology, hence limiting the design of and application of security rules. Our work uses a security ontology with a focus on the cybersecurity components to provide security services’ provisioning based on the reasoning capabilities and a model-driven methodology.

Finally, to conclude our analysis, Ekelhart et al. [23] proposed a framework for information security risk management to measure security through risk assessment, risk mitigation and evaluation. This included the presentation of a new methodology, AURUM, used to support the risk management standard using an ontological information security knowledge base to provide a consistent and comprehensive method for the risk manager. This proposal is limited in the sense that it focuses solely on risk management.

## 3. Proposed Cybersecurity Framework

As expressed in our hypothesis, a cybersecurity framework is an essential requirement for the complete adoption of the Internet of Things by industry, academia and domestic stakeholders. For that, it is needed to identify what are the security-relevant capabilities of the IoT devices in order to be connected and correctly used. Cybersecurity practices are vital to business and comprise a factor that has not yet been taken into consideration by companies as part of their risk management process [24].

**Figure 2:**
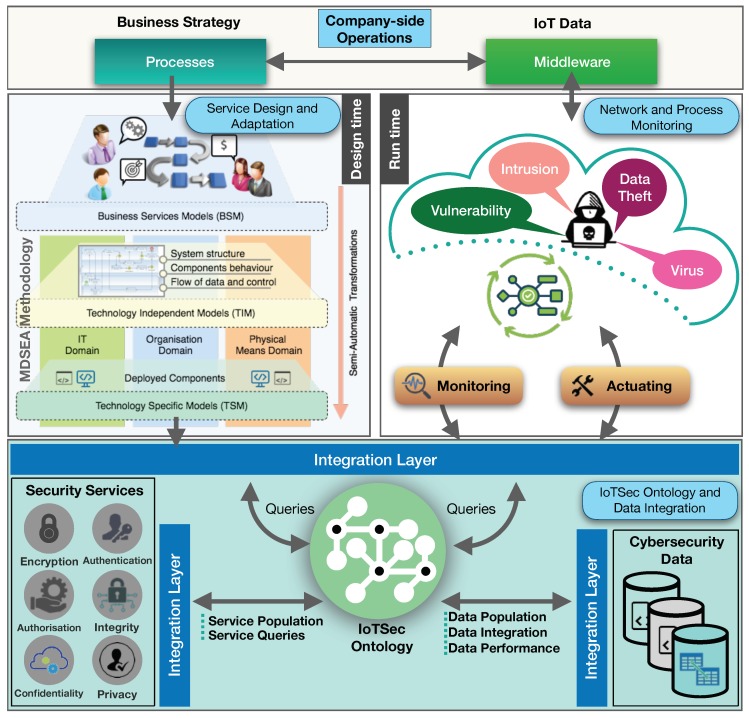
The proposed ontology-based cybersecurity framework.

This paper proposes an ontology-based cybersecurity framework focused on the security aspect of the Internet of Things. Our vision is to present a novel approach to improve the IoT cybersecurity focusing on the enterprise (company-side) monitoring, analysis and classification of security vulnerabilities in a knowledge base, while enabling the subsequent security service provisioning adjusted to the threats, hence improving security mechanisms around business processes and technology assets. For that purpose, the authors propose to separate the framework in three layers that deal with cybersecurity at design and run time, respectively, and an integration layer used by both (see Figure 2). At design time (top left side of the figure), the framework foresees the application of the MSDEA model-driven methodology to build and adapt existing security services semi-automatically reusing high-level of abstraction security service specifications in the development of technology-specific components. At run time (top right side of the figure), network and process monitoring mechanisms collect security alerts from different cybersecurity tools identifying and classifying situations of interest (e.g., threats and vulnerabilities) in a knowledge base formalised by the IoTSec ontology (integration layer, bottom part of the figure). Using such knowledge and its reasoning mechanisms, the ontology is able to propose suitable security services, which can or not be the ones specified at design time, adapting and actuating within IoT environments.

The company-side operations involve several business processes, which require data collection from IoT devices and sensors. Usually, these processes aim to perform parallel activities to achieve specific goals defined by the company in their business plan. The cybersecurity framework integrates the IoTSec ontology and data integration from distinct data sources into a knowledge base. This block provides data integration and population from the ontology information and the access of various security services regarding several business processes and network devices; requirements, ensuring security mechanisms against threats. The following sections describe these blocks in detail.

### 3.1. Service Design and Adaptation: Design Time Layer

Security services provide different types of protection for all company-side operations. Indeed, a system composed by IoT sensors only can be considered secure when security services ensure the different security properties (e.g., confidentiality, integrity, availability). At design-time, a company can decide to implement novel and specific services to address its business processes needs or adapt existing services already in place, which require adaptations to attend device constraints such as the implementation end-to-end security in the IoT, for which compressed IP Security Protocol (IPsec) provides a lower header overhead than link-layer security [25]. Using the run time layer modules and IoTSec, the cybersecurity framework supports minor changes in services such as the change of protocols or algorithms already designed. However, in the case of more profound adaptations that require new algorithms or protocols’ development, the design time layer is required, applying the MDSEA methodology to support the full-service life cycle, by generating code from high-level of abstraction specifications, hence accelerating the service design, adaptation and deployment time. With this approach, business users such as company managers can collaborate with developers and participate in the specification of the necessary security mechanisms.

The service design and adaptation is a block of the proposed framework to perform model transformations from a high-level business of abstraction to code artefacts in order to deploy new services according to environment requirements [26]. This approach considers distinct cartridges to make deployments on heterogeneous technologies. This block provides IoTSec a pool of security services (e.g., confidentiality, privacy, authorization, encryption, integrity and authentication) to be used in the network and process monitoring block. Each service is composed by a set of security mechanisms specifically designed to deal with each requirement. For instance, a security service for the encryption component requires the use of mechanisms, which provide data encryption/decryption. Such mechanisms include the use of secure protocols such as the Secure Socket Layer for secure communications.

### 3.2. Network and Process Monitoring: Run Time Layer

The network and process monitoring block provides two complementary methods to explore the environment: monitoring and actuation. The first method relies on the monitoring of situations of interest that are being analysed in each environment. This includes detection of possible intrusions, data theft, virus, ransomware, etc. Monitoring tools offer information regarding different types of security alerts that are then investigated using distinct security tools, such as firewalls, intrusion detection systems, vulnerability scanners, etc. Each situation is then analysed in order to identify whether there are suitable solutions (from the pool of security services in IoTSec) that can be applied in that specified moment to recover the system and improve cybersecurity. The second method focuses on such adaptations, i.e., to prevent threats in the business processes according to the security analysis results from the knowledge base. It consists of applying appropriate mechanisms or changing particular protocols to avoid detected security threats again.

This way, the run time layer of the proposed framework is responsible for identifying and classifying known threats in a knowledge base to offer appropriate security service to prevent future occurrences. A collection of monitoring tools provides information about intrusion detected in the environment and generates alerts using the IDMEF standard. When a situation of interest is identified, it is responsible for registering and classifying it within IoTSec according to its particular characteristics.

### 3.3. IoTSec Ontology and Data Integration Layer

The data integration layer provides cybersecurity information (e.g., threats and vulnerabilities) using the IoTSec ontology [27] with a pool of security already existing or generated using the design time layer and the MDSEA methodology. There are distinct cybersecurity data sources available that are included using an integration layer to provide data population, data integration and data performance from the queries on the ontology. Currently, the IoTSec ontology consists of certain statements, which are summarised in Table 1.

The integration layer of the proposed framework considers this knowledge base to perform the data integration with distinct data sources in a unified database. Using the ontology reasoning, the correlation between main classes of the ontology provides implicit information to offer suitable security solutions from services available in the integration layer. Service adaptations are required in some situations, then the framework uses the MDSEA methodology to transform a high-level of abstraction to minimize the service deployment. The integration layer provides information from pre-build cybersecurity services that can be external or developed at design time from service design and adaptation.

### 3.4. Design Time Usage

The design time usage requires a procedure before to start the proposed framework, which connects the IoTSec ontology with existing cybersecurity data sources using the Ontop framework [28] to provide data integration and population. However, after the data population, the design time layer may be applied before and after the network and process monitoring. The proposed framework provides support to the service development for situations that require generating of new algorithms or services’ functionalities that was not designed in the pool of security services. The design time layer tools can generate and provide new security services for the IoTSec pool.

### 3.5. Run Time Usage

The security monitoring capabilities are a feature of the proposed framework to identify and classify situations of interest (e.g., threats and vulnerabilities) in the environment. All monitoring tools used in the environment generate alerts from situations of interest such as known threats and intrusions. These alerts are analysed and classified according to the knowledge base. For each alert generated, queries are specified using the Protocol and RDF Query Language (SPARQL) [29] to check suitable security mechanisms to protect the asset or process vulnerabilities. In this context, our implementation uses the Protégé Editor [30] to process these queries and collect results from the IoTSec knowledge base. According to the results, one or more security mechanisms can be put in place, selected from the security services pool of the proposed framework.

### 3.6. Implementation Considerations

**Figure 3:**
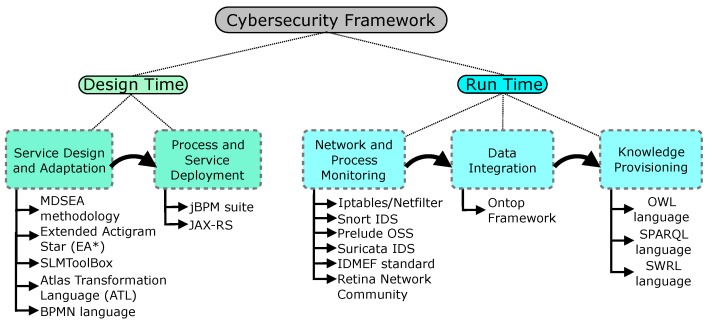
The logical relation of the application of implementation technologies and the proposed framework. BPMN, Business Process Model and Notation; IDMEF, Intrusion Detection Message Exchange Format; IDS, Intrusion Detection System; SWRL, Semantic Web Rule Language.

The implementation of our proposed ontology-based cybersecurity framework for IoT considers several technologies with the aim of achieving the requirement stressed in the concept. The implementation of the latter is described according to Figure 3: design time and run time.

At design time, there are two steps used to build and deploy security services: service design and adaptation and process and service deployment. The first explores the Model Driven Development using the MDSEA methodology to optimize the service development. The model transformation applied to the security management is described in detail in the previous work published by the authors in [26]. The business processes are firstly designed with the Extended Diagram Star ($EA^{*}$) technology in the SLMToolBox [31]. This diagram instantiates the Business Services Model (BSM) of the MDSEA methodology and allows one to represent the regular activities flow of a specific company or network that wishes to apply the cybersecurity framework. For specific implementation of new security services (or specific functionalities), the business model is transformed (using Atlas Transformation Language) into a Technology Independent Model (TIM) in Business Process Model and Notation (BPMN) language so that developers can improve it with specific technical details about data-related operations. In this phase, any deployment aspects are still disregarded, allowing one to focus only on the functionality. Finally, the TIM processes model is transformed to the TSM, where deployment issues are specified. The processes and service deployment step are responsible for the configuration of the the jBPM suite (Kie Workbench) [32] in order to perform and execute specific services in the Java API for RESTful Web Services (JAX-RS). These deployments enable the establishment of the designed security services within the business process. These services are finally made available to the knowledge base via integration of the IoTSec ontology, becoming available for future requests to address the same type of security issue.

At run time, there are three main steps responsible for the network and process monitoring, data population and knowledge provisioning from the IoTSec ontology. The monitoring step analyses business processes and technology assets to detect threats and vulnerabilities for the IoT system. It entails specific monitoring tools to identify threats such as Iptables/Netfilter [33], Snort [34], Prelude [35], Suricata [36] and vulnerabilities such as the Retina Network Community [37]. The security alerts generated from these monitoring tools follow the Intrusion Detection Message Exchange Format (IDMEF) [18]. Alerts raised are used to classify threats and vulnerabilities in the IoTSec ontology. The proposed framework uses SPARQL language to perform queries on the ontology to gather suitable information from potential threats in the IoT environment. The data integration step provides cybersecurity information from distinct data sources using the Ontop framework. This allows for the instantiation of a knowledge base from IoTSec ontology. The data population also uses Ontop to support data access through a conceptual layer rewriting the SPARQL queries (over the virtual RDF graph) to Structured Query Language (SQL) queries. Thanks to Ontop, the framework is capable of exploring the knowledge of different sources to provide data population, data integration and data performance. Finally, the knowledge provisioning from the IoTSec ontology uses the SPARQL language to perform queries and check all information in the knowledge base. Due to language flexibility, correlations between ontology classes can be used to cross information regarding the IoT environment.

## 4. Validation and Proof of Concept

This section describes the validation of the ontology-based cybersecurity framework. Section 4.1 presents the ontology validation with a methodology applying the Software product Quality Requirements and Evaluation (SQuaRE) standards [38] to identify its weaknesses and strengths. Section 4.2 presents an industrial scenario implemented in the frame of the EU C2NET project (http://c2net-project.eu) to verify the suitability of the application of our framework considering some cybersecurity issues of a real case.Please cite the link as a reference.

### 4.1. Ontology Assessment

**Figure 4:**
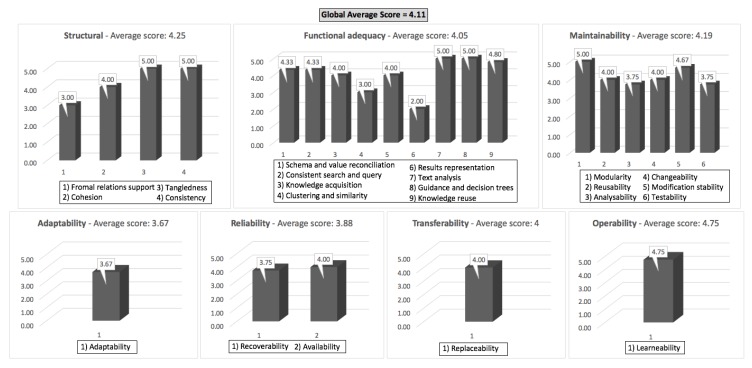
Average scores for the IoTSec ontology using OQuaRE metrics.

The ontology assessment covers the cybersecurity domain using the framework on basic components of security against potential vulnerabilities and threats. This evaluation follows a methodology. The adopted methodology for ontology evaluation was designed adapting the software engineering standard called OQuaRE framework [39]. This proposal was presented to help developers to identify weaknesses and strengths using a series of quality characteristics for ontologies according to SQuaRE standards. The model reuses the SQuaRE characteristics to the ontology evaluation, namely: structural, functional adequacy, adaptability, reliability, transferability, maintainability and operability. Figure 4 presents the evaluation scores obtained regarding the quality characteristics for ontologies:

- The structural characteristic consists of the formal and semantic ontological properties that are widely used in state-of-the-art evaluation approaches. It represents a complete cohesion with a good domain coverage. This helps to verify areas that are more closely connected. It reflects in the knowledge base as a result of extracting data from separate sources. Under structural characteristics, the relation of the number of properties and relationships presents a relatively low value to the formal relations support, which can be improved using inference rules to ensure better formal relations.
- The functional adequacy characteristic follows certain criteria according to the degree of accomplishment of functional requirements over different purposes. As its strengths, the evaluation showed consistent search and query and knowledge reuse considering the mean number of relationships associated, the number of properties per class and the length of the path from the leaf classes to the thing. Furthermore, the metric “mean number of properties per class” collaborates with the knowledge acquisition because it means that probably this ontology is more useful. However, a sub-characteristic demonstrated weaknesses associated with the number of instances. This aspect has no impact on the evaluation because the ontology requires a complete data population for an application in the real world.
- The adaptability characteristic checks if the ontology can be adapted for different specified environments without conducting other than those were identified for the ontology purpose. The metric “number of properties and relationships” is one essential factor to provide adaptability. This measure consists of a better understanding of how certain focal classes work. Hence, the number of relationships reflects the grouping within a class based on its relationship with other instances. However, a sub-characteristic affects the adaptation purpose of the ontology for distinct environments due to the largest path from the thing to a leaf class.
- The reliability characteristic matches the ontology maintenance of the level of performance under the stated conditions for a given period of time. The metric “maximum depth of the hierarchy tree from thing to a leaf class by the total number of paths” directly influences the availability sub-characteristic.
- The transferability characteristic presents the degree to which the software product can be transferred from one environment to another. The metric “number of properties and direct subclasses” allows one to adapt the ontology easily in another context. However, the metric “length of the largest path” affects the recoverability sub-characteristic because the higher is its score, then lower is the probability to recover.
- The maintainability characteristic provides the ability of ontologies to adapt to changes in the environment, in terms of requirements or functional specifications. The number of properties also impacts on reusability (of maintainability) because having a more precisely-defined ontology makes its knowledge more reusable.
- The operability characteristic harmonizes the knowledge necessary to use an ontology, and in the individual assessment of such use, by a single or a set of users. It is measured through the learnability sub-characteristic. The metric “number of the properties per class” reflects in the schema used at the instances level. This metric is a good indication of how well the use of information in the extraction process is. However, the maximum depth of the hierarchy tree from thing to a leaf class minimizes the effectiveness.

The results presented above demonstrate the strengths and weaknesses of the IoTSec ontology. It can be observed that the global average score has a value of 4.11. Particular quality metrics have affected some characteristic; still the general quality of our ontology is good. In accordance with the criteria of human evaluation, some future improvements could produce a better outcome.

### 4.2. Industrial Case Study to the Proposal Validation

**Figure 5:**
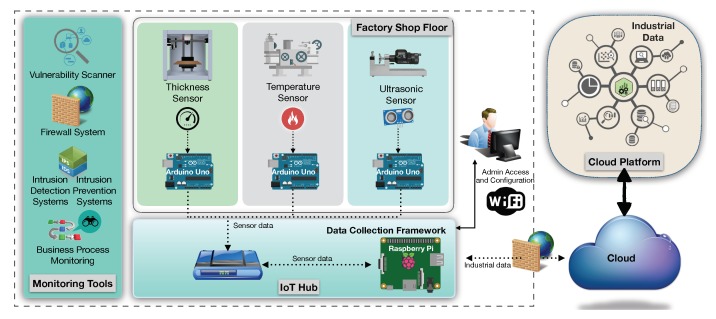
Industrial scenario in a factory shop floor.

In this section, a case study is used to validate the proposed cybersecurity framework for the IoT. This framework uses the IoTSec ontology as an intelligent support system to improve the cybersecurity of the environment. The manufacturing scenario of this industrial case study is a pilot from the EU C2NET project, which focused on improving the supply network optimization of manufacturing and logistic assets based on collaborative aspects, production and delivery plans. It aims to use smart devices to improve the production of product parts from different third-party organizations. The industrial scenario contains several sensors to provide IoT-based continuous data collection from supply network resources of the factory shop floor in a company of the metalworking industry, as depicted in Figure 5.

The IoT hub represented at the bottom refers to a device that performs the data management to be processed in the cloud platform using several cloud-based tools for supporting the supply network optimization and logistic assets based on collaborative demand. As a contribution, this cloud platform provides new ways to store relevant information securely from supply network partners in a public cloud. The cybersecurity management of this scenario, offers monitoring capabilities to detect and prevent threats within the shop floor networked environment.

#### 4.2.1. Process/Environment Monitoring

There are potential cybersecurity vulnerabilities of which malicious users could take advantage. These include unprotected communication channels, lack of an access control system, a private channel for sensitive information, and so forth. Furthermore, these aforementioned technologies contain known threats, as well as security mechanisms that are essential to ensure the presence of a secure environment. The Raspberry Pi 3 is one IoT device used in this industrial scenario (within the IoT hub) that has a variety of threats and vulnerabilities to be exploited by malicious users in the form of threats and other security hazards.

Some monitoring tools are responsible for identifying abnormal situations and generating security alerts such as the Intrusion Detection System (IDS), firewall system and vulnerability scanner. These alerts provide information to be analysed in the proposed framework. As each tool has a particular specification, in this work, the IDMEF format was adopted to make the interoperability between the monitoring tools and the framework.

#### 4.2.2. Ontology Usage for Monitoring

The proposed cybersecurity framework uses the IoTSec ontology and knowledge base to find suitable solutions and information over services according to generated alerts. In addition, the ontology uses the reasoning capabilities to identify the knowledge base uniformity, correctness of data instances and assertions using rules. This process derives implicit facts from the existing knowledge and can be classified into logic-based context reasoning, rule-based reasoning or deductive and inductive reasoning. Some ontology verification processes occur as a result of reasoning. These include:

- Verifying the consistency of the ontology and knowledge base.
- Verifying the unintended relationship between classes.
- Automatically classifying instances in classes.

The definition of inference rules is established with the Semantic Web Rule Language (SWRL) [40] with the Protégé editor using the reasoner Pellet [41] to make the rule processing. The reasoner manipulates the ontology logic using inference rules to reason with individuals, user-defined data types and debugging support for ontologies.

R1: hasPart(?x, ?y), hasPart(?y, ?z) → hasPart(?x, ?z)
R2: isSecurityMechanismOf(?sm, ?t), threatens(?t, ?v) → mitigates(?sm, ?v)
R3: protects(?sm, ?a), requires(?a, ?sp) → satisfies(?sm, ?sp)
R4: mitigates(?sm, ?v), threatens(?t, ?v) → isSecurityMechanismOf(?sm, ?t)
R5: SecurityMechanism(?sm), SecurityProperty(?sp), Threat(?t), affects(?t, ?sp), isSecurityMechanismOf(?sm, ?t) → satisfies(?sm, ?sp)

**Figure 6:**
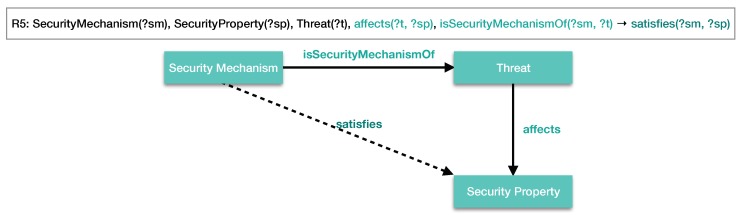
Graphical representation of the inference rule R5.

These inference rules presented above are methods, which allow new facts to be found from implicit knowledge. A graphical representation of a specific rule demonstrates the functionality to the knowledge base when the rule has a set of axioms that are valid. It fills an axiom that was implicit using true affirmation such as *X* is a security mechanism of *Y*, in the case of the axiom isSecurityMechanismOf(?sm, ?t). The inference rule R5 establishes a new relation when a set of axioms satisfies the rule requirements. Figure 6 represents this relation according to the rule.

Given the graphical representation of the inference rule R5, the object property isSecurityMechanismOf(?sm, ?t) provides the ability to link SecurityMechanism(?sm) and Threat (?t) and affects(?t, ?sp) for linking between Threat(?t) and SecurityProperty(?sp), respectively. Then, this association enables one to discover implicit facts from structured knowledge in the object property satisfies(?sm,?sp).

The knowledge reasoning has the ability to infer in several cases, discovering the relation among assets, vulnerabilities, threat security properties and security mechanisms. In the industrial scenario, several threats could violate information privacy due to bypassing security mechanisms. One of the common threats of industrial networks is the protocol used in a wireless LAN (WiFi protocol), especially for systems hosting valuable information. In this case, the best way to implement an effective level of security mechanism within a business organization is to utilize collaborative planning at a technical and organizational level with coordinated measures to ensure appropriate protection strategies. This is further stressed by the fact that single security mechanisms and separated tools cannot guarantee cybersecurity in a global setting. Threats are often conveyed by attackers in different ways, often categorised according to architectural layers. Each security threat affects one or more security requirements, and the system is protected using specific security countermeasures.

**Figure 7:**
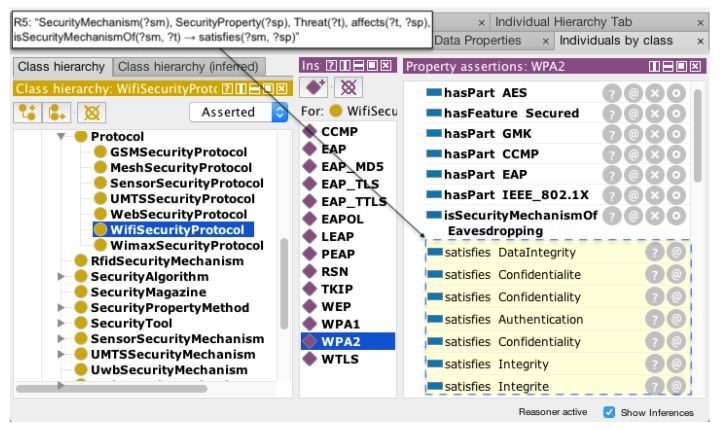
Results from the application of an inference rule.

Figure 7 presents the application of this rule in Protégé editor to produce inference results using the reasoner Pellet. The reasoning results from the rule R5 presents new facts (dotted line) regarding the object property satisfies according to the goal of the rule R5.

As reasoning results, the proposed framework provided a suitable security mechanism to prevent threats to the WiFi protocol called WiFi Protected Access, Version 2 (WPA2) that satisfies some instances of the *Security Property* class, ensuring protection of Authentication, Confidentiality and Integrity (these instances have distinct nomenclatures defined). According to the data collection, the system checks the knowledge base using the Protocol and RDF Query Language (SPARQL) [29] to identify suitable security mechanisms. SPARQL queries are used to obtain valuable knowledge of security attributes and individuals of the situation of interest in the IoT environment.

Within the scenario implemented, the proposed framework checked the knowledge base using SPARQL queries (Listing 1) to provide information about a situation of interest in that moment.

**Listing 1.** An example of query of the proposed framework.
  1 **SELECT** ?ASSET ?VULN ?THREAT ?SECPROP ?SECMEC_1 ?FEATURE_1

  2 **WHERE** {

  3  ?VULN iotsec:isVulnerabilityOf ?ASSET .

  4  ?VULN iotsec:isThreatensBy ?THREAT .

  5  ?THREAT iotsec:affects ?SECPROP .

  6  ?SECMEC_1 iotsec:isSecurityMechanismOf ?THREAT .

  7  ?SECMEC_1 iotsec:hasFeature ?FEATURE_1 .

  8  ?SECMEC_1 rdfs:label ?SMLabel .

  9  **FILTER regex** (?SMLabel, ‘WEP’)

 10 }


Listing 1 presents an SPARQL query with the association among vulnerabilities (?VULN), assets (?ASSET), threats (?THREAT), security property (?SECPROP), security mechanisms (?SECMEC_1) and features (?FEATURE_1) classes in Lines 3–8. In addition, this query filters (Line 9) the results for finding the only label with the expression “WEP”. This vulnerability of the Wired Equivalent Privacy (WEP) exposes weaknesses and requires suitable security solutions offered by the proposed framework.

Once a security issue in the environment is detected, the framework requests relevant information from this particular situation to the IoTSec ontology. The security alert reported the vulnerability *Unauthorized Access*, which affect two security properties, which are the Confidentiality and Integrity of the WiFi technology. As a consequence, several vulnerabilities could be exploited with the *Eavesdropping* threat.

**Figure 8:**
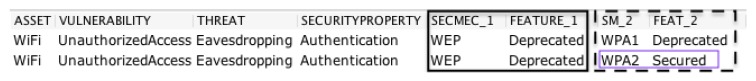
Results from the formal question to the cybersecurity framework.

Figure 8 presents the results from the formal question to the cybersecurity framework to identify alternative security protocols, which are suitable solutions to be deployed in this scenario. The query identified features of security mechanisms to offer on the environment. It only filters the results regarding its features to show an alternative mechanism with status *Deprecated*.

**Figure 9:**
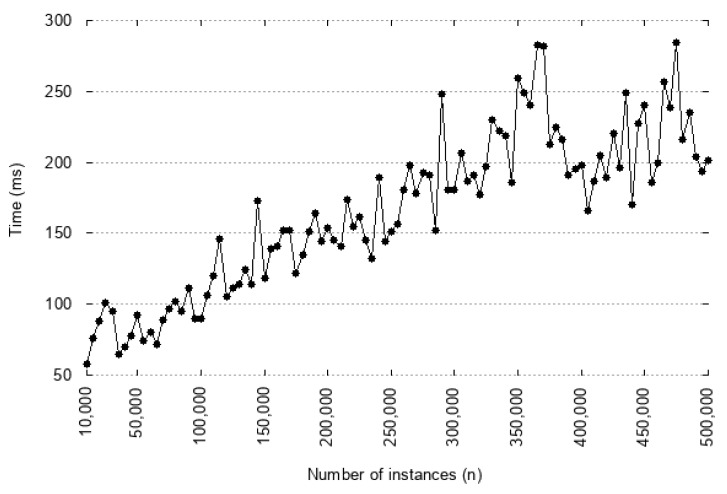
Processing time of a query with *n* instances in the results.

When implemented in this specific scenario, the query focused on vulnerable assets such as WiFi vulnerability, to provide a better level of security. To evaluate the costs present during the execution of the queries, 30 executions were performed with a different number of instances involved. Figure 9 presents the average time with different numbers of instances in the results. This evaluation shows that the processing time of queries increases according to the number of results. The execution time rises considerably with the number of instances (over 500,000). However, at 300 ms, the execution time does not appear to pose any problems to the industrial adoption of the IoTSec solution.

#### 4.2.3. Service Adaptation and Actuation on the IoT Network

Based on the SPARQL query, the ontology identified a service with a security mechanism: the WPA2 security protocol. This query presents protocol features from a previously-used protocol (continuous line) and a suitable solution (dotted line) using the object property *hasFeature* between classes *SecurityMechanism* and *Feature*.

In this context, the framework suggests the change of the wireless security protocol for secure communication over the computer network. Considering this, the proposed cybersecurity framework requires an adaptation of the wireless communication service provided by the service design and adaptation block. The adaptation of this service requires the instantiation of the WPA2 security protocol. This service was previously designed and instantiated with the WEP protocol at design time. Since this adaption is based on a mechanism only available in the repository of security services, the suggested solution does not require the generation process of a new service using the MDSEA.

The adaptation and actuation of this service provided a reconfigurable capability that allowed the security protocol to be changed in routers and IoT devices. This security protocol uses different stream ciphers and encryption schemes that offer better protection against unauthorized access. When implemented, the security improvement of this industrial scenario solved the vulnerability of the WEP protocol such as authentication forging, man-in-the-middle attacks and brute-force dictionary. This adaptation provided a much higher level of cybersecurity for IoT users and applications.

## 5. Final Considerations and Discussions

This paper introduced cybersecurity as the central challenge and one of the most important aspect to the complete adoption of the Internet of Things. The threat of exposing sensitive information from systems to the World Wide Web increased the complexity of the IoT cybersecurity. This is further aggravated by the risk of new threats and vulnerabilities regarding the heterogeneous connectivity of a high number of distinct IoT devices and systems. This paper proposed an ontology-based cybersecurity framework that addressed security concerns and increased protection of IoT devices and business processes of the Internet of Things. This involved the instantiation of an integrated technical framework using ontology and knowledge reasoning focused on the enterprise monitoring, security analysis, security service design and provisioning. The proposed cybersecurity framework was implemented and the IoTSec ontology instantiated and assessed with knowledge about known cybersecurity issues and the corresponding prevention measures, hence validating the hypothesis. The OQuaRE methodology achieved the global average score of 4.11 from a maximum of five.

As the strengths of the IoTSec ontology, the assessment identified an excellent consistency in its structure with an effective arrangement of the classes. In terms of functional adequacy, OQuaRE identified good individual average scores for knowledge acquisition and reuse. According to IoT cybersecurity, these characteristics associated with operability are essential to increase the amount and quality of information in the knowledge base. Nevertheless, the assessment also identified some weaknesses in the adaptability and reliability characteristics. Even though these characteristics were important in terms of usability, they are not relevant for the proposed framework because the proposed utilization of the IoTSec ontology does not consider changing of domains. One way to improve it would be to increase the number of data types and object properties and the average number of the direct parents as a form to create relations between different classes with more details for different domains.

The implemented industrial scenario addressed inherent challenges of IoT cybersecurity. The proposed framework established a knowledge base from distinct data sources using the IoTSec ontology to offer suitable security mechanisms according to known threats and vulnerabilities detected from the industrial environment. With the instantiated framework, the monitoring of assets was performed for different types of tools, collecting alerts from threats and vulnerabilities. These alerts were analysed to discover proper security mechanisms. This implementation conducted an accurate security analysis based on reasoning capabilities of the IoTSec ontology that determined some discoveries of unknown facts on the knowledge base. It also allowed finding solutions implicit between related classes of the ontology. Moreover, the results of the security analysis required adaptation of services, and the methodology adopted by the proposed framework provided support to accelerate the service design.

During the development of the proposed cybersecurity framework, some challenges were discovered concerning the devices’ features found in the industrial environment. The utilization of IoT devices with power and performance constraints created challenges in adopting traditional security services, and it required the adaptation of mechanisms with specific hardware characteristics. Furthermore, the actuation requires careful specification to avoid resource consumption such as lightweight security mechanisms.

Usually, zero-day threats or recent vulnerabilities identified from the anomaly-based intrusion detection system and vulnerability scan systems do not have security mechanisms published yet. In such cases, offering suitable solutions for “uncharted” behaviour is a hard task. A particular way to manage such situations is to establish standard actions according to a particular behaviour. However, the establishment of actions for each behaviour category makes it challenging to select effective mechanisms of different reasoning strategies under different categories of behaviours, which further stresses the challenge of uncertainty reasoning. In this context, Bayesian networks have been used to deal with ontology uncertainty, which requires probability determination in a structured form where each state is justified mathematically and takes into consideration specific real inputs.

### 5.1. Contributions of the Work

The proposal addressed in this paper provided a unified technical framework to monitor business processes and technology assets using an ontology and knowledge reasoning for the IoT cybersecurity domain. At this point, some contributions can be highlighted to improve security monitoring, analysis, as well as service design and provisioning to highlight particular asset constraints within an industrial environment. One of these contributions is the IoTSec ontology, which gathered cybersecurity knowledge about alerts and possible threats from the contextual information of security issues to correlate it with vulnerabilities and security properties. The correlation amongst classes provided links between basic elements of cybersecurity: assets, threats, security mechanisms, vulnerabilities and security properties. These links were fundamental in the finding of implicit data from the environment, particularly through reasoning capabilities from the ontology engineering.

Concerning run time security monitoring, generated cybersecurity alerts from different probe categories could be integrated using the IDMEF standard in the same knowledge base, providing integrated actuation services and checking mechanisms. The main contribution of this aspect was the minimization of problems with heterogeneous data from distinct security mechanisms used for intrusion detection and vulnerability scanners. The design and orchestration method provided capabilities to adapt security services already existing, or to generate a new one using the MDSEA methodology creating a pool of security services according to the particular needs of the application environment. Another contribution of this work was the aggregation of the run time monitoring and the design and orchestration method that offers suitable services based on the security requirements of the IoT cybersecurity.

### 5.2. Future Research Directions

Following the central challenge, i.e., IoT cybersecurity, there are still open research issues without ideal solutions. IoT cybersecurity is a severe problem for society as a whole, and several insights of future trends have the potential to deal with it. Digital platforms consist of a composition of technologies formed by components directly related to IoT cybersecurity such as 5G, edge computing and low cost communication. These technologies will create the most unpredictable and disruptive breakthroughs for humans. They fill the gap between the device sensors and data networks to provide awareness using back-end applications to the generated data from sensors. This interoperability is a requirement that prevents the emergence of broadly accepted IoT ecosystems [42].

Artificial Intelligence (AI) is a relevant field that has received much attention with the progress of IoT because it allows one to develop systems that learn, adapt and act autonomously to improve decision-making and business models within the digital market. This growth consists of several technologies such as decision trees, linear regression and neural networks, resulting in effective implementations of physical devices and services to deliver a new class of smart applications for business scenarios. This orchestration provides intelligent devices through the use of IoT platform services or models as-a-service from an adaptive perspective [43]. AI will offer better solutions for IoT cybersecurity that aim to identify threats even if it requires a short learning phase to establish which events are potential attacks. In our context, AI should provide alternatives to understand the system’ behaviour (e.g., to detect zero-day threats) and make sure that the situation of interest is happening. For instance, machine learning systems could be used in the future to analyse logs with statistical features to extract behaviour snapshots of the IoT network detecting threats and vulnerabilities from compromised IoT devices [44].

Ontologies provide a semantically-rich knowledge base for information management in several contexts such as business intelligence [45]. Ontology engineering is a key enabling technology to build a model of a specific domain, which has the capabilities to share a common understanding and to improve the communication between people and application systems [46]. In the cybersecurity domain, as explored in this article, ontologies support the automatic establishment of security metrics based on explicit and reasoning information about situations of interest and combined knowledge from multiple security experts. Furthermore, ontologies have improved the efficiency and effectiveness in security operations [47] and the natural language processing to help analysts to extract relevant pieces of information to characterize vulnerabilities and threats [48]. However, there are open issues that must be addressed to achieve a sufficient level of a multi-layered cybersecurity intelligence ontology to explore intelligence capabilities and understand potential threats against the ever-changing cybersecurity landscape.

Finally, the IoT is a fast-growing, increasingly complex network of connected sensors and devices. One important future approach to deal with real challenges is the adoption of a continuous adaptive risk and trust assessment, which allows real-time decision-making with adaptive responses [49]. Furthermore, the adoption of Software-Defined System (SDSys) is an approach to reduce the overhead in the control and management operations of complex computing systems such as Software-Defined Networking (SDN), proposed to eliminate the rigidity present in traditional networks [50]. It allows the softwarization of IoT infrastructure to improve the sensor networks’ agility and flexibility. This softwarization is provided with Software-Defined Security (SDS). Furthermore, SDS can provide a flexible and centralised security solution by abstracting the security mechanisms from the hardware layer to a software layer [51]. The aggregation of SDSys such as SDN and SDS will become one of the key transformations in 5G networks, which creates new opportunities to achieve SDN-based 5G network monitoring as an alternative to traditional network-wide monitoring initiatives [52,53]. The transition between traditional network architectures to SDN-based architectures is also an open issue. Most of these cybersecurity challenges of IoT applications are directly related to a centralised approach such as addressed in the SDN.

## Figures and Tables

**Table 1:** Number of classes, properties, axioms and annotations in the IoTSec ontology.

Ontology Metric	#	Ontology Metric	#
Classes	228	Logical Axioms	1895
Object Properties	24	Annotations	1418
Data Properties	7	Individuals	607
